# Who Wants to Become Italian? A Study of Interest in Naturalisation among Foreign Migrants in Italy

**DOI:** 10.1007/s10680-022-09639-y

**Published:** 2022-09-08

**Authors:** Elisa Barbiano di Belgiojoso, Livia Elisa Ortensi

**Affiliations:** 1 University of Milan-Bicocca, Milan, Italy; 2 Alma Mater Studiorum – University of Bologna, Bologna, Italy

**Keywords:** Naturalisation, Interest, Eligibility, Italy

## Abstract

Since the early 1990s, Italy, along with other countries situated at Europe’s periphery, has become an attractive destination for migrants due to its lax regulation of migration and its job market. Despite its restrictive naturalisation laws, an increasing number of migrants are becoming eligible for Italian citizenship, which has led to a growing number of naturalisations in recent years. Existing research exploring naturalisation and its determinants has found migrants’ ability to attain citizenship strongly depends on their interest in becoming a member of the host state, requirements (as defined by the host country), and their capacity to overcome various constraints such as the costs involved in the naturalisation process. However, few empirical studies have focused on immigrants’ interest in naturalisation. To fill this gap, we analyse migrants’ interest in naturalisation and how it correlates to their eligibility. This paper relies on the most recent data on interest in naturalisation from the 2018 and 2019 waves of the Regional Observatory for Integration and Multiethnicity of Lombardy (Italy). The results show that not all migrants are interested in naturalisation after assessing its perceived costs and benefits, thus confirming an instrumental approach to citizenship. Interest is mostly related to the legislation and conditions in the country of origin. Moreover, the relationship between eligibility and interest is highly dependent on civil stratification, and eligibility is not directly associated with interest.

**Supplementary Information:**

The online version contains supplementary material available at 10.1007/s10680-022-09639-y.

## Introduction

The naturalisation of foreign citizens is a recent and increasingly prominent issue in the Italian public discourse. In the early 1990s, countries at the periphery of Europe, and Italy in particular, became attractive to migrants due to those countries’ lax regulation of migration and labour force shortages. Before that period, migrants mostly entered Italy for reasons of opportunity rather than a genuine interest in the country and frequently migrated elsewhere after a short period (Fullin & Reyneri, 2011; King & De Bono, 2013; Reyneri, 1998). Although large-scale immigration can be considered a relatively recent phenomenon in Italy, five million of its residents—corresponding to 8.5% of the total population as of 1 January 2021—are currently foreigners (Istat, 2021a). Moreover, 63.1% of third-country nationals (TCNs) are long-term residents (as of 1 January 2020; Istat, 2020). Therefore, even though Italy’s standard procedure for TCNs requires they reside there for a decade to be eligible for naturalisation—one of the strictest requirements in Europe (Saurer, 2017; Vink & de Groot, 2010)—naturalisation is now becoming an option for many migrants finally meeting the requirements. Notably, over one million migrants acquired Italian citizenship between 2012 and 2019 (Istat, 2021b). If all eligible foreign residents were to acquire Italian citizenship, naturalised individuals would make up a non-negligible proportion of Italy’s citizen population. Nevertheless, the question remains: is migrants’ unconditional interest in naturalisation a likely scenario? Moreover, in general, how interested are migrants in naturalisation? What factors are most strongly correlated to interest in naturalisation?

While several studies exist on the naturalisation process and its advantages (e.g. Labussière & Vink, 2020; Peters et al., 2018, 2020), less is known about interest in citizenship acquisition. Naturalisation is the final result of a process that begins with the migrant’s interest and then depends on his/her capacity to overcome obstacles such as affording the costs associated with the naturalisation procedure and meeting the requirements established by the host country. Following the approach recently proposed by Huddleston (2020), we employ a cost–benefit approach to study interest in naturalisation in Italy to examine the relationship between interest, eligibility (i.e. meeting the requirements to apply for citizenship) and migrant status and then discuss the findings’ policy implications.

## Citizenship Acquisition: Interest and Eligibility

To the best of our knowledge, due to the lack of data, only a few empirical studies have explicitly analysed migrants’ interest in naturalisation (Anil, 2007; Huddleston, 2020; Kahanec & Tosun, 2009). Huddleston (2020) was the first to provide a clear definition of interest in naturalisation and a specific theoretical framework. In his pioneering study, he defines interest as ‘the perception of the desirability of formal membership and identification with the destination country’. He also stresses that interest in naturalisation reflects ‘migrants’ intended life plan’ (Huddleston, 2020, p. 5).

Although the literature rarely considers the role of interest in the naturalisation process explicitly, many scholars de facto discuss its determinants by acknowledging that migrants approach citizenship acquisition by carefully evaluating related advantages and costs (Bauböck, 2010; Jensen et al., 2019; Peters et al., 2016; Yang, 1994). Moreover, personal characteristics affect how people perceive naturalisation’s relative costs and benefits (Mazzolari, 2009) and ‘condition the perceived value and meaning of citizenship’ (Peters et al., 2016: 361).

Research has shown that naturalisation entails many benefits that could increase migrants’ interest. Becoming a citizen of another country may improve immigrants’ economic and socio-political integration, provide access to better jobs, expand their social networks, and increase their political participation (Gathmann & Monscheuer, 2020; Mazzolari, 2009; Pendakur & Bevelander, 2014). Some studies underline naturalisation’s advantages in terms of economic conditions, welfare benefits, mobility rights, and social status by theorising citizenship as ‘a portable good’. This approach is referred to as ‘instrumental’ or ‘strategic citizenship’ (Finotelli et al., 2018; Harpaz & Mateos, 2018; Ip et al., 1997) and assumes immigrants will be interested if a particular advantage is expected.

However, despite evidence of naturalisation’s benefits, not all migrants become citizens of the country in which they settle in once they meet the requirements (Finotelli et al., 2018; Peters et al., 2016; Sredanovic, 2014). Why do some eligible migrants elect to not naturalise?

According to Anil (2007), non-interested migrants do not perceive that there are sufficient advantages to becoming citizens. Likewise, in our view, civic stratification and rights granted by citizenship at birth among migrants are key to understanding that migrants’ interest in naturalisation is substantially driven by securing a stable residence and enhancing mobility rights. For migrants, especially in the early stages of the process, legal residence is a vital issue, as is employment (Della Puppa & Sredanovic, 2017; Morris, 2003). Like all EU member states, Italy recognises different legal statuses of belonging among foreigners, the most notable being national citizenship within the EU and European economic area (EEA) countries and TCN status (Morris, 2003). Undocumented TCNs, asylum seekers (i.e. people from unstable/insecure countries of origin) and TCNs holding fixed-term resident permits do not meet the naturalisation requirements. At the same time, they are expected to be interested in naturalisation due to the potential acquisition of a broader set of rights and privileges than those secured with their present status. Skulte-Ouaiss (2013) suggests that citizens from highly insecure and unstable areas consider dual citizenship a protection against insecurity in their countries of origin. However, migrants who already enjoy most of the benefits granted by citizenship (e.g. EU/EEA citizens or long-term residents) will be less interested because their current legal condition diminishes the relative advantage that would be achieved by naturalisation (Anil, 2007; Kahanec & Tosun, 2009; Peters et al., 2016; Vink et al., 2013).

In contrast to this instrumental approach, other recent studies explain interest or lack thereof by underlining the influence of identity and sense of belonging. These studies demonstrate that decision-making about naturalisation is not driven by ‘strategic choices’ alone (Donnaloja, 2020; Erdal et al., 2018). Sense of belonging remains an essential element in the decision; migrants are aware that, along with its advantages, naturalisation requires a significant commitment to integrate into the foreign society (Anil, 2007; Midtbøen et al., 2020).

In a similar vein, nation-specific restrictions on dual citizenship may affect migrants’ interest in becoming citizens; migrants whose country of origin does not allow dual citizenship would incur higher costs and fewer advantages by naturalising (Anil, 2007) and would therefore naturalise at a lower rate, as previous studies have corroborated (Harpaz & Mateos, 2018; Huddleston, 2020; Mazzolari, 2009; Peters et al., 2016; Vink et al., 2013, 2021; Yang, 1994). By contrast, migrants who plan to settle permanently and invest in their host country (e.g. by buying a home) will be more interested in naturalisation (Barbiano di Belgiojoso, 2016; Peters, 2019).

Another factor to be taken into consideration is eligibility. ‘Eligibility’ refers to the fulfilment of requirements for naturalisation, which may include maintaining sojourn for a certain period of time, developing language proficiency, providing proof of economic self-sufficiency, paying a naturalisation fee, providing evidence of not having committed certain crimes, and passing a knowledge test. Within Europe, there is considerably high variation in the strictness of such requirements (Huddleston, 2013; Jensen et al., 2019; Midtbøen et al., 2020; Schmid, 2020; Vink & de Groot, 2010; Vink et al., 2013). Previous studies (Labussière & Vink, 2020; Stadlmair, 2017) found that strict requirements reduce naturalisation rates; however, the relationship between eligibility and interest remains unclear and under-researched. Anil (2007) found that non-interested migrants were also frequently ineligible due to being unemployed and/or receiving welfare. According to Jensen et al. (2019), difficulties in meeting eligibility requirements could frustrate migrants’ desire to naturalise, but strong evidence of this connection has yet to be provided.

## Contribution of the Paper and Research Hypotheses

In our paper, we assess how interest in naturalisation varies among foreign migrants in Lombardy, a region in northern Italy, and analyse their perspectives on naturalisation requirements. We also describe self-declared reasons not to naturalise. Moreover, we aim to examine the determinants of interest in naturalisation and how eligibility and migrant status affects interest in naturalisation. To this end, we build on the ‘instrumental’ or ‘strategic citizenship’ approach to determine interest by assuming that high perceived costs or insubstantial benefits from naturalisation will deter interest.

Our first set of research hypotheses aims to assess the relationship between interest in naturalisation and aspects related to constraints and characteristics of the country of origin. We hypothesise that migrants regard the loss of citizenship in their country of origin as a high cost. Therefore, it is a solid deterrent to naturalisation:

### H1a:

Migrants whose naturalisation implies the loss of their original citizenship are less interested in naturalisation.

Migrants may also consider instability in their home country or restrictions associated with their current citizenship when considering dual citizenship:

### H1b:

Instability in the country of origin or restricted mobility rights granted by the citizenship of birth are positively associated with interest in naturalisation.

Second, we aim to disentangle the role of civic stratification and eligibility in shaping interest. Having a status (acquired by residence or attached to the citizenship of origin) that provides the possibility of unrestricted permanence in Italy and broad legal international mobility restricts the perceived benefits for migrants assuming an ‘instrumental’ or ‘strategic citizenship’ approach. Therefore, we hypothesise that migrants’ interest in naturalisation is lower for EU citizens and for non-EU citizens with a legal status that allows an unlimited stay.

### H2a:

Migrants whose resident status is already secure (EU citizens and long-term residents) are less interested in naturalisation than migrants without a secure status (fixed-term regular migrants, irregular migrants, or asylum seekers).

At the same time, based on the literature, we find that lack of eligibility may frustrate migrants; therefore, after controlling for the security of status, we hypothesise the following:

### H2b:

Lack of eligibility is negatively correlated with interest among migrants with a secure status.

As eligibility is determined by the requirements set forth in naturalisation laws, this hypothesis is highly relevant in the context of the recurrent political debate over possibly modifying those laws.

Finally, we consider the strength of migrants’ attachment to their host country, which can be strong (proxied by home ownership) or weak (proxied by short-term migration intention).

### H3:

Attachment to the country of immigration is positively related to interest in naturalisation.

## Acquiring Italian citizenship

### Requirements: the Role of Legislation

Italy is among the countries with a high level of bureaucracy within the naturalisation ordinary procedure in Europe (Huddleston, 2013).^1^ Vink and Bauböck (2013) classify Italy among ethnoculturally selective regimes. According to the MIPEX index’s ‘access to citizenship’ indicator, which examines countries’ naturalisation requirements and procedures, Italy, with an index equal to 40,^2^ ranked 34th in 2020 and is classified as “slightly unfavourable”. According to the Citizenship Law (CITLAW ordinary naturalisation indicator),^3^ Italy ranks 26th.

The current law for the acquisition of Italian citizenship dates back to 1992 (Law 91/1992), when Italy first started to embrace immigration. Briefly, this law establishes that:

1. A foreign-born citizen can acquire Italian citizenship after maintaining a continuous and legal presence in Italy for a period of 10 years or marriage to an Italian citizen.
2. A foreign-born child who lives in Italy acquires Italian citizenship from his or her parents once they have become citizens (lineal transmission).
3. A child born in Italy to foreign-born parents may apply for Italian citizenship once he or she comes of age, conditional upon continuous residence in Italy since birth (election).

It should be noted that, despite Law 94/2009 extending the residence requirement of residence from six month before marriage to two years after marriage and stipulating that applicants must remain married for a period of three years, marriage remains the fastest path to Italian citizenship (Finotelli et al., 2018). Moreover, refugees and EU citizens benefit from considerably lower residence requirements (five and four years, respectively).

Italy has repeatedly adjusted its laws, opting for more restrictive requirements aimed at ensuring migrants’ social and economic integration. In 2009, a naturalisation fee was introduced. Law 113/2018 further introduced a B1 language test,^4^ increased the fee from €200 to €250, and extended the maximum period it takes to process an application from 24 to 48 months. Italy’s requirement for duration of stay is among the strictest in Europe; in fact, only Switzerland requires a longer (12 years) sojourn (Saurer, 2017; Vink & de Groot, 2010). At the same time, it is essential to underline that Italy accepts dual citizenship.

### New Italian Citizens: a Review of Recent Data

**Fig. 1:**
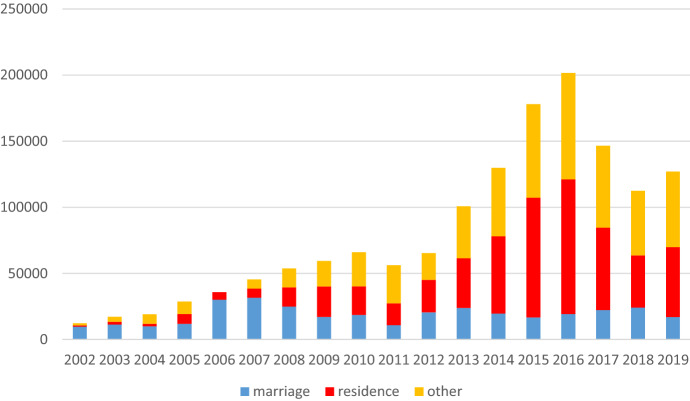
Acquisition of Italian citizenship in Italy from 2002 to 2019 by mode of acquisition. *Source*: Authors elaborations on data from the Ministry of Interior (2002–2010) and Istat (2011–2019)

The number of Italian citizenship acquisitions has increased considerably (Strozza et al., 2021). Since 2002, approximately 1.4 million foreigners have become Italians (see Fig. 1). Recent research shows this trend is primarily the result of ordinary naturalisation, transmission from parents to their children, and election. Marriage to an Italian citizen is less prominent than other mechanisms despite being the fastest route to citizenship (Bonifazi et al., 2017; Bonifazi, 2017) (Fig. 1). People’s paths to citizenship vary according to age at naturalisation; transmission from parents prevails among children while ordinary naturalisation and marriage are the most common pathways to citizenship among adults.

Among migrants who naturalised between 2012 and 2019, there is an equal gender distribution (49.2% are women). However, upon further inspection, a specific age profile emerges as there are higher concentrations of minors and adults than of young adults and older migrants (37.9% are minors, 18% aged 30–39 years and 20% aged 40–49 years) (Table 1).

**Table 1:** Naturalised migrants between 2012 and 2019 in Italy: composition by age and gender

Variable	%
*Age*	
Up to 19 years	37.9
20–29 years	10.1
30–39 years	18.8
40–49 years	20.6
50–59 years	9.8
60 + years	2.9
*Gender*	
Female	49.2
Number of naturalised	1,061,737

*Source*: Authors elaborations on data from Istat. Dati.istat.it

**Fig.2:**
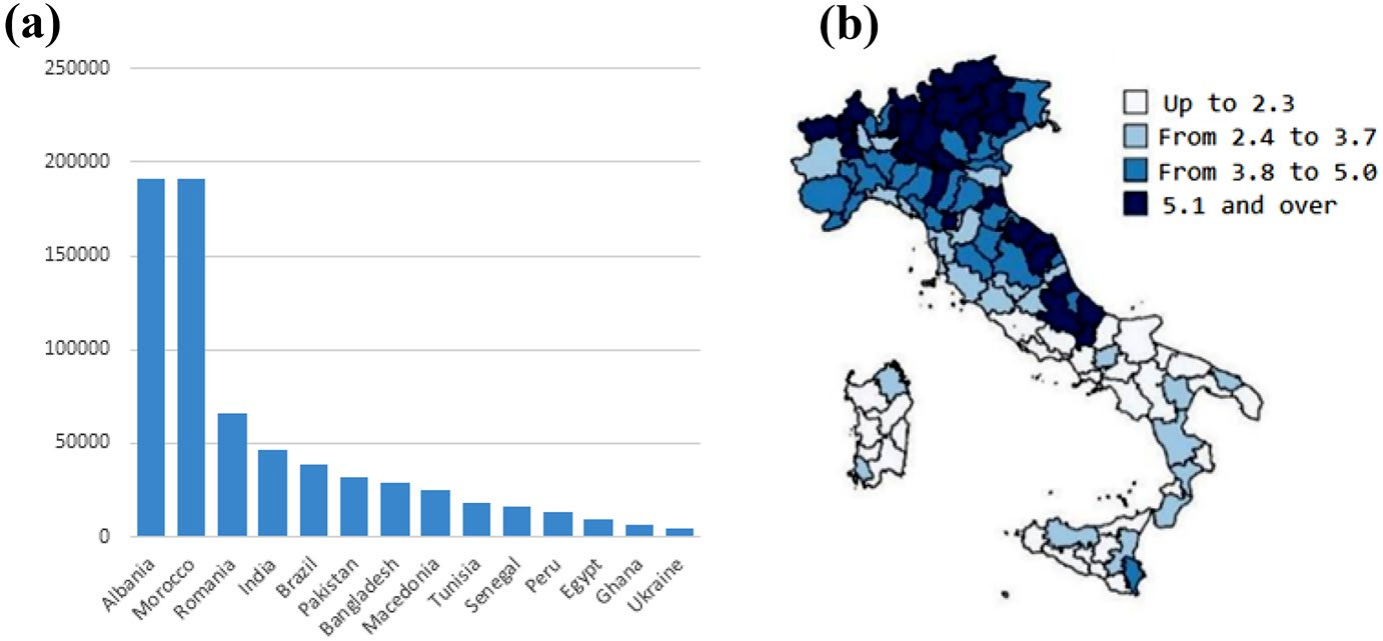
**a** Previous citizenship of naturalised migrants between 2012–2019 and **b** citizenship acquisition rate among non-EU resident citizens in Italy, 2017. *Source*: **a** Authors elaborations on Istat data; **b** Blangiardo (2019)

The naturalisation rate also varies considerably by country of origin. As shown in Fig. 2a, most naturalised citizens between 2012 and 2019 immigrated from Albania and Morocco while a smaller number came from Romania and India. Consistent with the geographic pattern of long-term settlement observed in Italy, naturalisation tends to occur in the northern and central regions of the country, as shown in Fig. 2b. More specifically, 27% of migrants naturalised between 2012 and 2018 resided in Lombardy.

As aforementioned, depending on the country of origin, naturalisation implies both advantages and disadvantages for immigrants. EU and EEA citizens are always allowed free circulation and access to the labour market while TCNs fall into various subgroups with different conditions for their admittance, residence, and citizenship acquisition. After five years of legal residence, TCNs can apply for EU long-term resident status.

Table 2 shows an indicator of the so-called passport power (Passport Index; Sect. 5 for details). The gap between the power of an Italian passport and that of other passports provides a measure of the gains in terms of mobility rights each nationality can expect. Table 2 also describes the dual citizenship policies of the most common countries of origin among migrants in Italy. Loss of citizenship from the country of origin is among naturalisation’s most notable disadvantages.

**Table 2:** Passport Index and the possibility of dual citizenship. Most common countries of origin among migrants in Italy, 2018

	Country of origin	Passport Index Italy = 164	Dual citizenship
1	Romania	157	Allowed
2	Albania	109	Allowed
3	Morocco	67	Allowed
4	China	77	Not allowed
5	Ukraine	130	Allowed
6	Philippines	69	Allowed
7	India	66	Not allowed
8	Bangladesh	44	Not allowed
9	Moldova	111	Allowed
10	Egypt	55	Allowed

*Source*: Passport Index: https://www.passportindex.org/ and dual citizenship according to the variable dual citizenship binary of the MACIMIDE Global Expatriate Dual Citizenship Dataset: https://macimide.maastrichtuniversity.nl/dual-cit-database/ Vink, Maarten; De Groot, Gerard-Rene; Luk, Ngo Chun, 2015, “MACIMIDE Global Expatriate Dual Citizenship Dataset” 10.7910/DVN/TTMZ08, Harvard Dataverse, V5 [2020]

## Data and Methods

### Data

A lack of data is the primary reason for the scarcity of studies on interest in naturalisation. As highlighted by Huddleston (2020), who uses data from selected European cities, survey data on interest in and requirements for naturalisation are not available at the national level, and Italy is no exception. For this reason, we focus on the data available for the Italian region of Lombardy. A unique, regularly updated dataset providing information on foreigners is available due to the Regional Observatory for Integration and Multiethnicity of Lombardy (ORIM) conducting face-to-face retrospective multipurpose surveys annually since 2001. The survey focuses on migrants aged 18 or above from all countries, except former EU15 and European Free Trade Association (EFTA) countries, the USA, Canada, Australia, New Zealand, and Japan living in Lombardy. This includes undocumented migrants and naturalised citizens (Blangiardo, 2018). The survey is based on centre sampling (Baio et al., 2011) and designed explicitly to be representative at the regional level. Researchers have repeatedly used ORIM surveys in ground-breaking studies on migrants’ legal status when nationwide survey data were unavailable (e.g. Dustmann et al., 2017; Fasani, 2015).

Likewise, we utilise a pooled dataset from the 2018–2019 ORIM surveys. Since we analyse interest in naturalisation at the moment of the interview using cross-sectional data, we need to exclude migrants who have already been naturalised from the analysis. Indeed, if we can easily infer that all naturalised migrants were interested and eligible at a specific time, we cannot measure their socio-economic characteristics before naturalisation. Our data allow us to examine the interviewees’ personal and family characteristics (e.g. legal status, homeownership, number of children) only at the time of the interview, which cannot be assumed to reflect the interviewees’ situation before naturalisation. Moreover, naturalisation may have driven changes in their socio-economic conditions. The final subsample we used for this study consists of 2372 foreign migrants.

To model potential costs and benefits of naturalisation related to country-level characteristics, we include measurements of the origin countries’ levels of development, political stability, and toleration of dual citizenship. Each country’s level of development is measured using the Human Development Index (HDI, 2019), which is a summary measure of three critical dimensions of human development: a long and healthy life, access to knowledge, and a decent standard of living. The index provides a scale ranging from 0 to 1, where a higher score indicates a higher level of development.

Political instability is measured through the ‘index of political instability and violence’, which measures the likelihood of violent threats to or changes in government (e.g. terrorism). The higher the index, the more stable the country (World Bank, 2020).

The Passport Index measures so-called passport power—how different passports affect their holders’ identity, opportunities, mobility, and overall quality of life. The higher the index, the greater the passport power (Arton, 2018).

### Variables

We use data on migrants’ self-declared interest in naturalisation for our analysis. Following Huddleston (2020), we define two measures for interest in citizenship as dependent variables, although we adapt his definition to our data.^5^ We use a ‘broad measure of interest in citizenship’ (see Models 1 and 2), coded 1 = ‘*Interest’* if the migrant declares an interest in citizenship acquisition or has applied for citizenship, regardless of his or her eligibility, and 0 = ‘*No interest*’ (reference category) otherwise.

To check robustness, we use a ‘narrow measure of interest in citizenship’ on a subsample that excludes from the analysis migrants that have already applied for citizenship (see Models 3 and 4) coded 1 = ‘*Interest’* if the migrant declares an interest in citizenship acquisition and 0 = ‘*No interest*’ (reference category) otherwise.

These two definitions allow us to consider and partially understand and control the process of selectivity in naturalisation that emerges as a substantial issue when using cross-sectional data and is likely to impact the results.

As independent variables, we consider some essential demographic characteristics of migrants:

- *Gender* ‘Female’, ‘Male’ (reference category);
- *Age at arrival* (in years) and its square;
- *Education level* ‘None or elementary’ (reference category), ‘Secondary or tertiary’.
- *Years since migration* (in single years). Notably, this variable measures the time spent in Italy, including any eventual irregular spell. This is not, in other words, the legal length of stay, which can be considerably lower.

Following the literature, we add two variables to describe family structures:

- *Presence of minor children cohabiting in Italy* ‘No’ (reference category), ‘Yes’;
- *Respondent has a partner* ‘No’ (reference category), ‘Yes’.

To account for attachment to the destination country, we consider:

- *Homeowner status* ‘No’ (reference category), ‘Yes’.

We consider both self-declared eligibility and a variable combining legal status and eligibility (self-declared eligibility and security of status):

- *Self-declared eligibility for naturalisation* ‘No’ (reference category), ‘Yes’.
- *Self-declared eligibility and security of status* ‘Non-eligible undocumented migrant or asylum seeker’; ‘eligible long-term resident or EU citizen’; ‘non-eligible long-term resident or EU citizen’; ‘eligible migrant with a fixed-term permit’; and ‘non-eligible migrant with a fixed-term permit’.

Finally, we also control for the year of the survey.

To measure the effect of the country of origin, we consider two variables. The first variable, labelled *Dual citizenship allowed in the country of origin,* [‘No’ (reference category), ‘Yes’], assesses the possibility of having dual citizenship according to the country of origin’s law, as reported in the MACIMIDE Global Expatriate Dual Citizenship Dataset. The second variable, labelled *socio-economic and political stability*, is obtained from a principal component analysis (PCA) applied to three variables:

1. Human Development Index (HDI);
2. Passport Index;
3. Political Instability Index.

The component extracted measures the country of origin’s economic and political stability and its degree of development. The higher the value, the greater the country’s stability and development.

### Method and Analytical Strategy

We fit two random-intercept logistic regression models using different measurements of ‘eligibility’. Model 1 includes self-declared eligibility while Model 2 substitutes self-declared ‘eligibility’ with ‘self-declared eligibility and security of status’.

Migrants with the same citizenship share the same costs and benefits regarding the possibility of holding dual citizenship, the difference in passport power compared to Italian natives, and the political instability of the country of birth. Given the dichotomous nature of the variables accounting for interest in naturalisation, we fit a set of multilevel models for clustered dichotomous responses.

We used a random-intercept logistic regression to relax the assumption of conditional independence among the interest in naturalisation among migrants (i) of the same nationality (j). We include citizenship of origin-specific random intercept $\zeta_{j}$, which represents the combined effect of all omitted citizenship/country of origin-specific covariates

$${\text{logit}} \left\{ {\Pr \left( {y_{ij} = 1|x_{ij} ,\zeta_{j} } \right)} \right\} = \beta_{1} + \beta_{2} x_{3ij} + \beta_{2} x_{3ij} \ldots + \beta_{n} x_{nj} + \zeta_{j}$$

where $\zeta_{j} |x_{ij} \sim N\left( {o,\psi } \right)$ and $\zeta_{j}$ are independent across citizenships *j.*

### Robustness and Consistency Checks

As robustness and consistency checks, we estimate Models 3 and 4 excluding migrants who have already applied for citizenship, reducing the subsample size to 1777 cases and using the narrow measure of interest in citizenship as the dependent variable. The rationale is to highlight the effect of self-selection on naturalisation in the analysis of interest when only cross-sectional data are available.

Model 3 includes self-declared eligibility while Model 4 substitutes self-declared ‘eligibility’ with ‘self-declared eligibility and security of status’. Based on Model 4, we estimate predicted probabilities, the results of which are included in Appendix.

We use an alternative variable to homeownership to indicate a solid attachment to the destination country, namely short-term migration intention (weak attachment): ‘Stay in Italy’ (reference category), ‘Onward migration’, and ‘Return migration’ (see Models 1bis-4bis). We define short-term migration intention as self-declared migration intention within 12 months of the interview. The results are also included in Appendix.

In addition, we verify the stability of our results using cross-validation. This method splits the data randomly into *k* partitions. For each partition, the specified model is fit using the other *k*-1 groups and the resulting parameters are used to predict the dependent variable in the unused group. Finally, to check if a specific migrant group drove the results, we use the same models while deleting one migrant group after another and comparing the results. The results of these additional checks^6^ are stable both in terms of values and significance.

## Results

### Descriptive Results

Table 3 shows the distribution of the sample.

**Table 3:** Descriptive statistics of the sample

Variable	Categories	%
Gender	Female	48.5
	Male	51.5
Education	None or elementary	40.0
	Secondary or tertiary	60.0
Minor children	Yes	46.1
	No	53.9
Having a partner	Yes	67.4
	No	32.6
Double citizenship allowed by country of origin	Yes	75.8
	No	24.2
Short-term migration intention	Stay	90.4
	Onward	5.6
	Return	4.0
Homeownership	Yes	18.7
	No	81.3
Year of the survey	2018	40.1
	2019	59.9
Eligibility and security of status	Non-eligible undocumented and asylum seeker	6.3
	Eligible long-term resident and EU citizen	42.4
	Non-eligible long-term resident and EU citizen	21.0
	Eligible migrant with fixed-term permit	6.2
	Non-eligible migrant with fixed-term permit	24.1
Mean age at arrival (in years)	25.5 (sd. 9.9)	
Mean length of stay (in years)	12.4 (sd. 7.2)	
*N* (unweighted)	2372	

*Source*: Own elaboration on ORIM data pooled dataset 2018–2019

The lengthy bureaucratic procedure and the strictness of the residence requirement indeed explain why migrants delay acquiring Italian citizenship and naturalise at a relatively low rate. However, the lack of interest in naturalisation also plays a role. As shown in Table 4, data collected for Lombardy reveal that 20.2% of foreign migrants are not interested in becoming Italians. Interestingly, eligibility does not seem to affect interest: eight migrants out of ten are interested in becoming Italian regardless of their eligibility. Our data confirm the strictness of eligibility criteria in Italy, showing that half of the foreign migrants with foreign citizenship are ineligible (51.5%). Despite being eligible for naturalisation, a small group (9.4%) is not interested in acquiring Italian citizenship. In contrast, 40.6% of migrants are interested in naturalisation but do not yet meet the requirements. Moreover, it should be noted that only 51% of eligible migrants have applied for naturalisation.

**Table 4:** Interest in Italian citizenship and eligibility among migrants with foreign citizenship

Eligibility	Broad interest in citizenship			*N*
	No	Yes	Total	
No	20.7%	79.3%	100.0%	1249
Yes	19.5%	80.5%	100.0%	1123
Total	20.2%	79.8%	100.0%	2372
*N*	433	1939		

Years 2018 and 2019

*Source*: Own elaborations on ORIM surveys for 2018 and 2019

Table 5 highlights the high variability across countries of origin in the two dimensions of analysis. Migrants from Bangladesh and Morocco are the most likely to be interested in naturalisation. Meanwhile Chinese and Romanian migrants have the lowest interest rates despite a high percentage of them being eligible. In the case of Chinese migrants, the Chinese government’s ban on holding dual citizenship is the most likely explanation. In contrast, Romanian migrants are probably deterred by their EU status and high passport power, which reduces the perceived benefits of naturalisation.

**Table 5:** Interest and eligibility to acquire Italian citizenship among migrants by country of origin. Years 2018 and 2019

Country of origin	Interested (%)	Eligible (%)	*N*
Albania	88.7	60.7	108
Romania	45.9	67.4	83
Ukraine	74.3	42.8	97
Moldova	77.8	44.7	43
Bangladesh	96.2	48.8	57
China	33.9	50.6	120
Philippines	75.5	48.1	83
India	89.9	51.6	84
Egypt	82.6	42.2	220
Morocco	90.7	54.9	184
Total subsample	79.8	51.5	2372

*Source*: Own elaborations on ORIM surveys for 2018 and 2019

Our data allow us to analyse the self-declared motivation to apply or not apply for Italian citizenship. The primary considerations involved in deciding whether to pursue naturalisation are, on the one hand, avoiding the bureaucratic procedures necessary for a residence permit and, on the other, securing the opportunities granted by Italian citizenship, such as freedom of movement and advantages for family members (see Table 6). Notably, these advantages apply only to non-EU migrants, as post-2003 EU citizens can settle and work in Italy and within the EU without restrictions. Only a small group of migrants report ‘feeling Italian’ as their motivation (5.3%). Similarly, ‘acquiring civil rights’ is rarely mentioned as a motivation (1.2%). These results indicate that migrants generally approach Italian citizenship instrumentally rather than as a means to substantiate an existing sense of belonging.

**Table 6:** Motivation for applying, or not, for Italian citizenship

Interested in citizenship		Disinterested in citizenship	
Motivations	%	Motivations	%
No more problems with bureaucracy	43.5	Satisfied with the current situation	33.0
I could guarantee advantages to my family	23.6	I have a fixed-term migratory project	15.4
Mobility and work within Europe	18.3	Loss of native citizenship	13.7
Feeling Italian	5.3	Not feeling Italian	13.7
Work in public administration and access to competitive exams	3.5	I will never meet the requirements	1.6
Acquisition of civil rights	1.2	Other reasons	22.5
Other reasons	4.7		
*N*	513	*N*	182

Year 2018

Source: Own elaborations on the ORIM survey for 2018

The lack of interest in naturalisation is chiefly related to satisfaction with one’s present legal status, the impossibility of holding dual citizenship, engagement in a short-term migration project, or identity (not feeling Italian). It should be noted that among those migrants reporting that they are ‘not interested despite being eligible’, the requirement to renounce their native citizenship is a major determinant of uninterest.

### Multivariate Results

The results of the random-intercept logistic regression models allow us to identify the characteristics associated with interest in the naturalisation net of control variables (see Table 7).

As found by Huddleston (2020), our research confirms that essential socio-demographic characteristics are not significantly associated with interest in naturalisation.

As expected, the perceived cost of losing one’s citizenship of origin is highly relevant for migrants. The possibility of holding dual citizenship strongly correlates with migrants’ interest in becoming citizens (H1a). High passport power, political stability, and high HDI in the country of origin correlate negatively with interest in naturalisation (H1b). In contrast, migrants from the most unstable and less developed countries are the most interested in naturalisation. Attachment to the destination country, as proxied by home ownership, is positively correlated to migrants’ interest in naturalisation (H3), partially confirming previous results (Peters, 2019).

**Table 7:** Odds ratios and standard error of random-effects logistic regression models with the dependent variable broad interest in naturalisation (reference category ‘no interest’)

	Broad interest	
	Mod. 1	Mod. 2
Female (ref. male)	1.152 (0.153)	1.158 (0.156)
*Education (ref. none or elementary)*		
Secondary or tertiary	1.158 (0.151)	1.171 (0.130)
Minor children (ref. no)	0.849 (0.130)	0.837 (0.130)
Having a partner (ref. no)	1.117 (0.183)	1.218 (0.203)
Age at arrival	0.998 (0.023)	0.997 (0.023)
Age at arrival squared	1.000 (0.000)	1.000 (0.000)
Double citizenship allowed by the country of origin (ref. no)	3.010** (1.027)	3.250*** (1.096)
Homeowner (ref. no)	1.627** (0.303)	1.558* (0.290)
Years since migration	1.060 (0.036)	1.073 (0.040)
Years since migration (squared)	0.997* (0.001)	0.997* (0.001)
Eligibility (ref. no)	1.084 (0.158)	
Socio-economic and political stability	0.697*** (0.065)	0.697*** (0.065)
*Eligibility and security of status (ref. non-eligible and fixed-term)*		
Non-eligible undocumented migrant or asylum seeker		2.095* (0.647)
Eligible long-term resident or EU citizen		1.404 (0.279)
Non-eligible long-term resident or EU citizen		1.160 (0.228)
Eligible migrant with a fixed-term permit		0.696 (0.176)
Year of the survey	Yes	Yes
Constant	Yes	Yes
Rho	0.163	0.168
Sigma *u*	0.801	0.817
LR test of rho = 0	chibar2(01) = 146.59 Prob > = chibar2 = 0.000	chibar2(01) = 144.01 Prob > = chibar2 = 0.000
*N*	2249	2241

*p < 0.05; **p < 0.01; ***p < 0.001

*Source*: Own elaborations on the ORIM data from the pooled dataset for 2018–2019

The overall length of stay, which our data record without any distinction between legal and illegal presence, is not a good proxy for eligibility. Length of stay has a limited and nonlinear effect on interest in citizenship. This should be read considering the specificity of the Italian migration model characterised by high irregular migration at arrival, especially from certain areas of origin.

The link between eligibility, legal status, and interest in naturalisation is highly dependent on the subpopulation analysed and migrant status. Considering eligibility, we found a positive but non-significant relationship with interest in citizenship. However, eligibility intertwines with migrant status. To clarify this result further, we re-estimate Model 1 combining eligibility with migrants’ security of status (Model 2). Results show that non-eligible undocumented migrants and asylum seekers are more interested in becoming Italian than non-eligible migrants with a fixed-term permit. In addition, eligible migrants with a secure status are just as interested in citizenship as non-eligible migrants with a fixed-term permit or non-eligible undocumented and asylum seekers. At the same time,^7^ eligible migrants with a secure status are more interested than eligible migrants with a fixed-term permit. Thus, the results do not support Hypothesis 2a because once we control for the country of origin’s characteristics, migrants with a secure status are not less interested in becoming citizens than all other migrants.

**Fig. 3:**
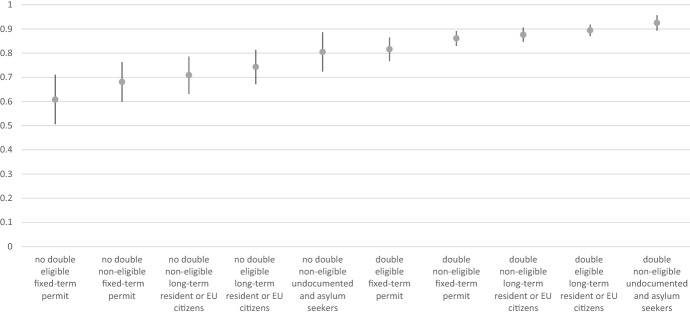
Predicted probabilities and confidence intervals of broad interest estimated based on Model 2. Non-overlapping bars indicate statistically significant difference at *p* < 0.05 level (Goldstein & Healy, 1995). *Source*: Own elaborations on the ORIM data from the pooled dataset for 2018–2019

It should be noticed that the intertwining of eligibility, possibility of holding dual citizenship, and migrant status determine different levels of interest. Accordingly, the benefits correlated to one of these dimensions should be compared to the eventual costs of the others. Figure 3—reporting predicted probabilities estimated for Model 2—shows that the puzzle is quite complex. The top and the bottom of the raking clearly distinguish two groups of migrants. At the same time, in the middle of the ranking defined by predicted probabilities, we observe that individuals with different combinations of eligibility, dual citizenship, and security of status frequently have similar chances of being interested in naturalisation. Figure 3 shows that undocumented migrants and asylum seekers with the possibility of holding dual citizenship are the most interested in becoming citizens. Naturally, given their present legal condition, they would obtain the highest benefits from naturalisation.

Contrary to our expectation (H2b), lack of eligibility does not frustrate migrants’ interest in citizenship among migrants with a secure status. Eligible migrants with a secure status (long-term resident permit or EU citizenship) from countries that allow dual citizenship are highly interested in citizenship. The same is observed for non-eligible migrants with a secure status and the possibility of dual citizenship. This group is composed of migrants with a long-term resident permit (at least five years of regular residence in Italy) who might therefore qualify for citizenship within a few years. This condition makes citizenship a feasible goal and allows them to eventually complete their original migratory project, which might include obtaining a powerful passport (Della Puppa & Sredanovic, 2017).

Non-eligible migrants with a fixed-term permit and the possibility of dual citizenship top the list of the most interested parties. The advantages of acquiring Italian citizenship for this group are considerable, e.g. mobility within Europe, elimination of bureaucratic procedures, and the possibility of working in another European state.

At the opposite end of the ranking, we find migrants from countries not allowing dual citizenship comprising migrants with a fixed-term permit regardless of their eligibility and non-eligible migrants with a long-term resident permit or EU citizenship. Both groups have high naturalisation costs (loss of citizenship) and limited benefits, especially migrants with long-term resident permits and EU citizens who already possess most rights except for the right to vote in elections.

There is a clear relationship between the impossibility of holding dual citizenship and the lack of interest in naturalisation, as declared by migrants who have to renounce their native citizenship to become Italians. Conversely, eligibility does not affect migrants’ interest in citizenship.

### Robustness Checks

This analysis excludes foreign migrants who have already applied for citizenship, thus selecting a subpopulation of eligible migrants who are mostly uninterested in naturalisation. Our results show a significant negative relationship between interest and eligibility (see Models 3 and 4 in Appendix). A second selection-driven dichotomisation consistently appears according to eligibility as well as the country of origin’s effect (see Fig. A1 in Appendix). Non-eligible migrants are more interested in citizenship than eligible migrants with differences according to their legal status (because interested migrants have, in most cases, already applied). Undocumented migrants and migrants with fixed-term permits are the most and least interested in becoming Italian citizens, respectively, while migrants with a long-term resident permit or EU citizenship show average level of interest. The results for the population not subjected to self-selection because they are not eligible holds true across models. Model 3, which ‘artificially’ excludes ‘soon-to-be citizens’, suggests how using cross-sectional data on foreign migrants may lead to biased results due to self-selection. Indeed, among eligible non-applicants, a negative relationship with eligibility exists, but this is the result of the impact of self-selection on application and naturalisation of interested migrants.

If we consider attachment to Italy, the results are consistent with Model 1 when we replace the variable ‘homeownership’ with the variable ‘short-term migration intention’ (see Appendix Models 1bis-4bis). The results confirm the strong positive association between attachment to the host country and interest in naturalisation, as indicated by short-term migration intention and interest in naturalisation (H3). Naturally, migrants intending to return permanently to their country of origin within 12 months of the survey are less interested in naturalisation. Among these migrants, a short-term migration project is among the most common self-declared reasons for the lack of interest in acquiring citizenship. Similarly, migrants expressing a short-term onward migration intention (i.e. a permanent relocation to a third country within 12 months of the interview) are less interested in naturalisation than those who declare a settlement intention.

## Conclusion

This paper analyses citizenship acquisition among foreign migrants in Italy, focusing on an under-researched issue in literature: migrants’ interest in naturalisation and its relationship with eligibility. No recent data on migrants’ interest in naturalisation are available at the national level; therefore, we examine evidence from the northern Italian region of Lombardy, for which data are available. This area is an interesting case study for multiple reasons. First, Lombardy hosts approximately a quarter of the foreign population living in Italy. The region offers broader job opportunities than other areas, generating an important internal flow of foreigners from southern to northern Italy. Permanently settled migrants (i.e. those with a regular presence, higher duration of stay in Italy, higher naturalisation rate, and family presence) are more prevalent in northern Italy compared with the rest of the country (Blangiardo, 2019; Cremaschi & Devillanova, 2020; Istat, 2020). Accordingly, Lombardy’s naturalisation rates are consistently higher than the overall national rate. While generalisation of our results to Italy is not among the study’s goals, we are aware that an *ad-hoc* survey at the national level could help understand whether the results for Lombardy could be representative of the entire country.

Our analysis negatively answers our initial question, ‘Is migrants’ unconditional interest in naturalisation a likely scenario?’ Indeed, approximately 20% of migrants are not interested in becoming Italian, and even some of them, despite being eligible, are uninterested. Our data show that despite the high percentage of migrants interested in acquiring Italian citizenship (79.8%), 15 years after their arrival, only approximately 25% of migrants living in Lombardy had actually acquired it.^8^ Thus the naturalisation rate in Lombardy is considerably lower than those of some central and northern European countries, such as Sweden, the Netherlands, Denmark, France, and Belgium (Huddleston, 2020; Vink et al., 2021). This gap can be attributed to the stricter requirements and procedural obstacles highlighted by previous studies (Huddleston, 2013, 2020; Strozza et al., 2021). Indeed, as of 2018–2019, more than half of the foreign population living in Lombardy still do not meet the requirements.

Moving on to our second query, what factors are most strongly correlated to interest in naturalisation? Our results show that, in line with a previous study, personal characteristics do not affect immigrants’ interest in citizenship (Huddleston, 2020). Rather, the country of origin’s citizenship laws and stability and migrants’ security of status affect the perceived costs of naturalisation and subsequent interest. Migrants approach citizenship instrumentally: the higher the gains, the higher their interest in naturalisation; conversely, the higher the losses, the lower their interest in becoming Italian. EU citizens and migrants from countries with high levels of economic and political stability have limited gains. In contrast, migrants with precarious legal statuses, e.g. undocumented migrants and asylum seekers, whose mobility is restricted by their current passport and legal status are more likely to be interested in citizenship. The loss of the citizenship of origin is also an extreme cost as it is strongly related to identity and is, therefore, negatively associated with interest in naturalisation. Conversely, attachment to Italy and economic investment in the host country increases migrants’ interest in becoming citizens.

Crucially, self-declared eligibility is not in itself correlated with interest. However, our results suggest that the relationship between eligibility and interest is biased by self-selection’s effect on the application process and consequent naturalisation and should therefore be studied using panel surveys.

Our paper highlights two critical points from both the political and theoretical perspectives. First, interest in naturalisation is indeed widespread in the Lombardy region. However, it cannot be assumed that all migrants are interested in naturalising. Second, the weak role of eligibility in shaping interest is particularly relevant for the political debate. While strong attention is usually attributed to legislation in the immigration destination, our data suggest that legislation, stability, and security conditions of the countries of origin play a much more substantial role in shaping interest. The possibility of holding dual citizenship seems to be a precondition for many migrants: renouncing the citizenship of their country of origin may impact one’s identity and sense of belonging to one’s native country. It may also cause the loss of political and economic rights in the country of origin.

Our paper has some limitations, most of which are related to the data’s cross-sectional nature. As observed for welfare state provisions (de Jong & de Valk, 2020), the relevance of naturalisation might change throughout one’s life (e.g. after childbirth or regularisation). Eligibility and interest are not fixed and may change over time depending on plans, security of residence status, and growing attachment to the country of settlement. This is a crucial point that our study can only partially address. Panel data would be essential to better understand the evolution of interest in naturalisation over time since migration increases the chance of new and fluid conditions that might shape interest.

Moreover, the ORIM surveys lack specific information on belonging and identity that could help disentangle the relationship between these dimensions and the instrumental approach to citizenship.

Despite these limitations, these results are of particular interest considering the lack of evidence-based analysis on drivers of naturalisation in Italy and in light of the recurrent public debate over potential modifications to the current law. In 2017, an intense debate took place in Italy over whether to change the current law based on the *jus sanguinis* principle. The proposed change aimed to relax requirements only for Italian-born second-generation children of long-term resident parents (*ius soli*) and children who had completed part of their formal education in Italian schools^9^ (*ius culturae*). After centre-left parties entered the second government led by Conte in September 2019 and the government led by Draghi in February 2021, activists and others within civil society renewed the calls for reform. However, the naturalisation law has thus far remained unchanged and is unlikely to change in the near future. Our paper suggests that despite the high relevance attributed to eligibility in the Italian debate, migrant legislation and conditions of the country of origin are most crucial determinants of interest in becoming an Italian citizen rather than the fulfilment of requirements.

## Supplementary Information

Below is the link to the electronic supplementary material.

Supplementary file1 (DOCX 34 KB)

## Data Availability

Not available.

## Footnotes

1 The eight CITIMP bureaucracy indicators measure: (1) power of authority to confirm, check, and decide on application; (2) number of times that documentation is checked in procedure; (3) existence of inter-agency systems for document-checking; (4) number of deciding authorities for naturalisation; (5) type of deciding authority; (6) expertise of deciding authority; (7) maximum legal time limit for procedure; (8) scope and sanctions for time limits.

2 Source: http://www.mipex.eu/access-nationality.

3 Source: Global Citizenship Observatory (http://globalcit.eu/citizenship-law-indicators/).

4 Applicants who have signed an integration agreement or hold EU long-term resident status are exempted from certification (EMN, 2020).

5 Our broad definition of interest partially differs from Huddlestone’s (2020) because we do not include naturalised migrants in our model due to consistency reasons, as explained in Sect. 5.1.

6 The results are available upon request.

7 We estimate all possible comparisons according to the categories of the variable self-declared eligibility and security of status.

8 This proportion is calculated on the whole sample, including naturalised migrants.

9 For further details on the political debate, see Pasetti 2019.

## Code Availability

Available upon request.
