# Supplementary material for: Who Wants to Become Italian? A Study of Interest in Naturalisation among Foreign Migrants in Italy
Source: Eur J Popul. 2022 Sep 8;38(5):1095–118. doi: 10.1007/s10680-022-09639-y (PMC9726997; doi:10.1007/s10680-022-09639-y)
Supplement: Supplementary file 1 — Supplementary file1 (DOCX 34 KB) [file 10680_2022_9639_MOESM1_ESM.docx]

**Appendix**

**Robustness checks**

**Table A1** Robustness check: narrow interest. Odds ratios and Standard Error of random-effects logistic regression models with the dependent variable narrow interest in naturalisation (reference category ‘no interest’)

| Variables | Narrow interest | |
| --- | --- | --- |
|  | Mod.3 | Mod. 4 |
| Female (ref. Male) | 1.211 (0.173) | 1.223  (0.176) |
| Education (ref. none or elementary) |  |  |
| *secondary or tertiary* | 1.103 (0.152) | 1.115  (0.155) |
| Minor children (ref. No) | 0.804  (0.133) | 0.795  (0.132) |
| Having a partner (ref. No) | 1264  (0.222) | 1.341  (0.239) |
| Age at arrival | 1026 (0.026) | 1.024  (0.026) |
| Age at arrival squared | 0.999  (0.000) | 0.999  (0.000) |
| Double citizenship allowed by the country of origin (ref. No) | 3.010*** (0.944) | 3.068***  (0.972) |
| Homeowner (ref. No) | 1.025  (0.213) | 1.016  (0.210) |
| Years since migration | 1.033  (0.035) | 1.047  (0.039) |
| Years since migration (squared) | 0.998  (0.001) | 0.997*  (0.001) |
| Eligibility (ref. No) | 0.453***  (0.067) |  |
| Socio-economic and political stability | 0.725***  (0.065) | 0.726***  (0.065) |
| Eligibility and security of status (ref. non-eligible migrant with fixed-term permit) |  |  |
| *non-eligible undocumented migrant or asylum seeker* |  | 1.932*  (0.594) |
| *eligible long-term resident or EU citizen* |  | 0.556**  (0.112) |
| *non-eligible long-term resident or EU citizen* |  | 2.231  (0.240) |
| *eligible migrant with a fixed-term permit* |  | 0.422**  (0.112) |
| Year of the survey | YES | YES |
| Constant | YES | YES |
| Rho | 0.135 | 0.137 |
| Sigma u | 0.720 | 0.723 |
| LR test of rho=0 | chibar2(01) = 96.58 Prob >= chibar2 = 0.000 | chibar2(01) = 94.89 Prob >= chibar2 = 0.000 |
| N | 1,691 | 1,685 |

Legend: * p < 0.05; ** p < 0.01; *** p < 0.001

Source: Own elaborations on the ORIM data from the pooled dataset for 2018–2019.

**Table A2** Robustness check: weak attachment to host country, broad and narrow interest. Odds ratios of the random-effects logistic regression model with the dependent variable interest in naturalisation (reference category ‘no’).

|  | Broad interest | | Narrow interest | |
| --- | --- | --- | --- | --- |
|  | Mod. 1bis | Mod. 2bis | Mod.3bis | Mod. 4bis |
| Female (ref. Male) | 1.172  (0.166) | 1.180  (0.170) | 1.195  (0.183) | 1.200  (0.186) |
| Education (ref. none or elementary) |  |  |  |  |
| *secondary or tertiary* | 1.214  (0.168) | 1.217  (0.171) | 1.106  (0.183) | 1.112  (0.166) |
| Minor children (ref. No) | 0.833  (0.137) | 0.812  (0.136) | 0.725  (0.129) | 0.713  (0.128) |
| Having a partner (ref. No) | 1.261  (0.205) | 1.262  (0.226) | 1.308  (0.248) | 1.380  (0.264) |
| Age at arrival | 0.989 (0.024) | 0.986  (0.024) | 1.020  (0.027) | 1.018  (0.027) |
| Age at arrival squared | 1.000  (0.000) | 1.000  (0.000) | 1.000  (0.000) | 1.000  (0.000) |
| Double citizenship allowed by the country of origin (ref. No) | 3.239**  (1.108) | 3.479**  (1.189) | 3.389***  (1.093) | 3.526***  (1.152) |
| Short term migration intention (ref. Settlement) |  |  |  |  |
| *onward* | 0.542*  (0.141) | 0.510*  (0.134) | 0.438**  (0.122) | 0.413**  (0.116) |
| *return* | 0.159***  (0.042) | 0.151***  (0.040) | 0.167***  (0.046) | 0.159***  (0.044) |
| Years since migration | 1.047 (0.042) | 1.053 (0.042) | 1.022  (0.038) | 1.030  (0.042) |
| Years since migration (squared) | 1.000  (0.000) | 1.000*  (0.000) | 0.998  (0.001) | 0.998  (0.001) |
| Eligibility (ref. No) | 1.119  (0.247) |  | 0.443***  (0.070) |  |
| Socio-economic and political stability | 0.702**  (0.067) | 0.697***  (0.067) | 0.714***  (0.067) | 0.709***  (0.067) |
| Eligibility and security of status (ref. non-eligible and fixed-term permit) |  |  |  |  |
| *non-eligible undocumented or asylum seeker* |  | 2.309*  (0.780) |  | 3.600***  (1.398) |
| *eligible long-term resident or EU citizen* |  | 1.585*  (0.332) |  | 2.367***  (0.463) |
| *non-eligible long-term resident or EU citizen* |  | 1.288 (0.275) |  | 0.722  (0.202) |
| *eligible migrant with a fixed-term permit* |  | 0.710  (0.184) |  | 1.689*  (0.361) |
| Year of the survey | YES | YES | YES | YES |
| Constant | YES | YES | YES | YES |
| Rho | 0.156 | 0.163 | 0.132 | 0.135 |
| Sigma u | 0.782 | 0.800 | 0.708 | 0.716 |
| LR test of rho=0 | chibar2(01) = 123.57 Prob >= chibar2 = 0.000 | chibar2(01) = 121.58 Prob >= chibar2 = 0.000 | chibar2(01) = 83.49 Prob >= chibar2 = 0.000 | chibar2(01) = 81.98 Prob >= chibar2 = 0.000 |
| N | 2,029 | 2,022 | 1,510 | 1,505 |

Legend: * p < 0.05; ** p < 0.01; *** p < 0.001

Source: Own elaborations on ORIM data of the pooled dataset for 2018–2019.

Fig. A1 Robusteness check: narrow interest. Predicted probabilities and confidence intervals of narrow interest estimated based on Model 4

Note: Non-overlapping bars indicate statistically significant difference at p < 0.05 level (Goldstein and Healy, 1995).

Source: Own elaborations on the ORIM data from the pooled dataset for 2018–2019.
